# Key Attributes and Clusters of the Korean Exercise Healthcare Industry Viewed through Big Data: Comparison before and after the COVID-19 Pandemic

**DOI:** 10.3390/healthcare11152133

**Published:** 2023-07-26

**Authors:** Sung-Un Park, Deok-Jin Jang, Dong-Kyu Kim, Chulhwan Choi

**Affiliations:** 1 Department of Sports and Health, Hwasung Medi-Science University, Hwaseong-si 18274, Republic of Korea; psu@hsmu.ac.kr; 2 Department of Sports Medicine, Shinhan University, Uijeongbu-si 11644, Republic of Korea; tmzlqn@shinhan.ac.kr; 3 Department of Sports Science, Chungwoon University, Hongseong-gun 32224, Republic of Korea; 4 Department of Physical Education, Gachon University, Seongnam-si 13120, Republic of Korea

**Keywords:** exercise, healthcare, technology, COVID-19

## Abstract

This study aims to predict the characteristics of the exercise healthcare industry in the post-pandemic era by comparing the periods before and after the coronavirus disease 2019 outbreak through big data analysis. TEXTOM, the Korean big data collection and analysis solution, was used for data collection. The pre-pandemic period was defined as 1 January 2018–31 December 2019 and the pandemic period as 1 January 2020–31 December 2021. The keywords for data collection were “exercise + healthcare + industry”. Text mining and social network analysis were conducted to determine the overall characteristics of the Korean exercise healthcare industry. We identified 30 terms that appeared most frequently on social media. Four common (*smart management*, *future technology*, *fitness*, and *research*) and six different clusters (*sports education*, *exercise leader*, *rehabilitation*, *services*, *business*, and *COVID-19*) were obtained for the pre-pandemic and pandemic periods. *Smart management*, *future technology*, *fitness*, and *research* are still important values across both periods. The results provide meaningful data and offer valuable insights to explore the changing trends in exercise healthcare.

## 1. Introduction

The healthcare industry is a promising sector that is growing rapidly in a social environment that greatly values healthy lifestyles [1]. According to a 2013 report on healthcare by Deloitte [2], the paradigm of healthcare has changed from the healthcare 1.0 era (which focused on the prevention and spread of infectious diseases) and the healthcare 2.0 era (which focused on diagnosis and treatment) to the healthcare 3.0 era (which focuses on prevention and management) [3,4].

In the healthcare 3.0 era, customized services can be provided to individuals based on advanced information and communications technologies (ICTs), thus enabling the realization of new and diverse services [5]. Additionally, in the healthcare 3.0 era, interest and participation in exercise and sports have gained momentum, indicating that prevention and management are highly valued [6]. In the healthcare 3.0 era, new, advanced technologies are appearing in the form of convergence, and changes in the exercise environment due to the coronavirus disease 2019 (COVID-19) and new exercise trends are being presented. In order to understand the exercise healthcare industry, reviewing exercise healthcare elements from various perspectives is important. According to Deloitte’s Healthcare Industry Report 2018 [7], the healthcare industry currently needs a strategic shift from volume (expanding scale) to value (value creation).

Technological developments bring many amenities to society and serve as a driving force for social development; however, the key aspect of the participation of regular physical activity in daily life is the leveraging of ICTs for health promotion [8]. During the COVID-19 pandemic, public interest in health increased, while non-face-to-face culture began to take root. Therefore, the exercise paradigm shifted from offline to online activities [9]. In the case of home training regimes such as Peloton (Peloton Interactive Inc., Manhattan, NY, USA), exercise healthcare can be implemented through an offline-to-online service that links existing offline activities to online modalities [10]. Moreover, wearable devices and mobile applications collect data on physiological indicators (e.g., sweat secretion, respiration rate, and body temperature) and provide information regarding recommended exercise methods and the optimal amount of exercise [11,12]. Accordingly, device development and service-related industries required for exercise healthcare are expected to continue to expand.

Global companies such as Apple, Amazon, Facebook, Google, and Microsoft have declared their entry into the healthcare industry and invested approximately 37 trillion won [13]. Similarly, Kakao, a major ICT company in Korea, announced that it will advance into the new market centered on sports within the healthcare field in the future by promoting innovation that focuses on sports healthcare through its subsidiary, Kakao VX [14]. As the exercise healthcare industry emerges, a new academic approach based on scientific methods to broadly understand this novel market should be adopted.

This study attempts to predict the emerging characteristics of the exercise healthcare industry in the upcoming post-COVID-19 era by comparing the periods before and during the pandemic through big data analysis. The research questions set to achieve the purpose of this study are as follows:

1. What are the key attributes related to the exercise healthcare industry during the COVID-19 pandemic?
2. What are the values discovered in relation to the exercise healthcare industry during the COVID-19 pandemic?

## 2. Literature Review

### 2.1. Exercise Healthcare Industry

Exercise healthcare aims to provide customized services for the improvement of individuals’ health by combining health promotion and maintenance through exercise with ICT technology [15]. Early exercise healthcare focused on fragmentary functions that provided information generated during exercise, such as exercise time, steps, calories, and heart rate [16]. Currently, it guides customized exercise programs considering individual health conditions (such as physical fitness level, nutritional status, body composition, blood pressure, and blood sugar), systematically manages posture and motion targets in real time, and provides overall health management services [17].

Exercise healthcare records the effects of daily life (such as exercise, stress, and sleep), on the heart and evaluates the risk of diseases such as high blood pressure, three-way fibrillation, and sleep apnea through these heart rate data [18]. In this process, customized exercise programs are guided in real time through artificial intelligence and machine learning technology, and body information is measured after exercise and provided to users and medical staff [19].

The Korean healthcare market, which is the backdrop of this study, is expected to grow from 3 trillion won in 2014 to about 14 trillion won in 2020 [20]. As a result of examining actual users, it can be seen that the global rate has more than doubled from 17% in 2013 to 42% in 2020 [21]. Between 2019 and 2021, the number of users increased by 40%, reaching 56 million in 2021 [21]. The public’s interest in health has increased due to the COVID-19 pandemic, with the spread of online culture intensifying participation in exercise healthcare.

### 2.2. Big Data Analysis

Big data analysis collects and refines online data, enabling exploratory research on the market based on the relevance of keywords [22]. Big data research has the advantage of allowing consumer-oriented studies, as it enables the collection and analysis of social media data generated by users or interested persons in related fields [23].

Observing and recording the thoughts and actions of members of society is one of the key elements in discovering the basis of social science [24]. In the era of big data, all information in life is produced, measured, recorded, and stored. It is differentiated from the data collected for existing social science research in that it is not created separately by artificial intervention but is created “naturally” and reflects reality [24]. With these changes, the causal relationship, which has been an ideal criterion for establishing the framework of social science research, has been transformed into a correlation [25]. Correlation as a realistic alternative to efficiently analyze rapidly produced and accumulated data further strengthens its position [26]. In other words, big data can potentially diagnose the problems of current society and further predict those of future society through precise observation of current social phenomena, future forecasting power, and mutual comparison [27]. Therefore, it is necessary to approach big data as a complex construct that includes data and the patterns of production and consumption surrounding them rather than simply as a technological phenomenon in the narrow sense of new, quantitatively and qualitatively different new data. Additionally, this phenomenon must be scrutinized within the framework of social science: the theory–method–data interaction [24].

The utilization and analysis of big data have a tremendous impact on various fields such as healthcare research. As analysis techniques using big data are gradually being developed, they are used in research and analyses [28,29,30]. According to Farhadloo et al. [31], a big data analysis of the Zika virus spread was able to predict the expression trend. Additionally, the European Union (EU) is using big data analysis results as the main basis for establishing national policies [32]. Korea particularly has an excellent network infrastructure and generates a large volume of data, thus providing favorable conditions for big data analysis [33,34]. Several studies have shown that physical and emotional health can be promoted through exercise [35,36]. Furthermore, exercise healthcare provides users with customized services merging exercise and ICTs; however, as the user-centered service environment is rapidly changing [37], examining exercise healthcare’s elements (i.e., exercise, technology, and health) from more diverse perspectives is necessary. In other words, exercise health care services may vary depending on the development and application environment of new technologies as well as changes in awareness of exercise participation due to pandemics such as COVID-19 [1,2,3,4].

## 3. Materials and Methods

### 3.1. Data Collection and Refinement

TEXTOM V6.0 software (The IMC Inc., Daegu, Republic of Korea), the Korean big data collection and analysis solution, was used for data collection because it enabled the collection and analysis of social data on the Korean exercise healthcare industry. Atypical texts that appeared on news, web pages, and blogs on NAVER and Google [38,39], which are the most used portal sites in Korea, were also collected. For data collection, the pre-pandemic period was defined as 1 January 2018 to 31 December 2019 and the pandemic period as 1 January 2020 to 31 December 2021. The keywords for data collection were “exercise + healthcare + industry”.

In this study, data collection identifies and clarifies the type of information we seek. The scope of the data to be collected, then, needs to be limited to the characteristics of the keywords. Data refining refers to a process to convert unstructured test data to a structured format [40]. During data refining, Korean mono-syllabic parts of speech were deleted because these did not represent the correct meaning. Table 1 presents the data collection procedure.

### 3.2. Research Procedure

The research procedure was analyzed by applying big data analysis. First, the data were collected and refined using TEXTOM. A modified version of FullText Software (developed by Professor Loet Leydesdorff at University of Amsterdam., Amsterdam, Netherlands) is TEXTOM, which is a user friendly data analysis solution through text mining technology, (a) collecting data, (b) refining data, and (c) processing matrix data generation in the Korean web environment [23,41]. It is a useful software (TEXTOM V6.0) by the Korea Information and Communication Technology Association [42] and is currently being used in various research published from the National Research Foundation of Korea [43]. Second, From the refined data, the text-mining analysis using (a) frequency and (b) term frequency–inverse document frequency (TF-IDF) analysis extracted the top 30 terms. Text mining is an analytical method that extracts meaningful information based on useful patterns and relationships in unstructured text data [44]. Following the frequency analysis, the inverse document frequency (IDF) emerged, making it possible to verify the importance of the terms more efficiently.

**Figure 1:**
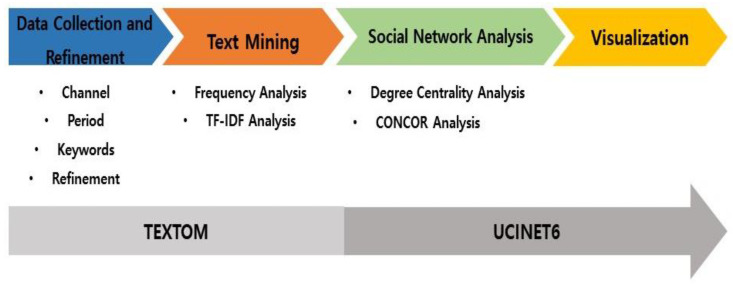
Research procedure.

Third, social network analysis (SNA) can analyze the meaning and pattern of a message and the relationship between the realization of ideas and words used simultaneously in a sentence without assuming a specific table of contents [45]. SNA is primarily used in the field of social science to derive significant implications for relationships within networks [46]. Therefore, this study identified the degree structure among terms and conducted a network analysis between terms related to the exercise healthcare industry through the Netdraw function using UCINET 6 (Analytic Technologies Corp., Lexington, KY, USA). UCINET 6 implements the relationship between individual words and the overall structure in three dimensions through visualized data and is useful for modeling keyword phenomena [47]. Additionally, a CONCOR analysis was conducted to derive clusters of similar terms related to the exercise healthcare industry. Finally, the derived data were visualized using tables and figures. The details of this procedure are as Figure 1.

### 3.3. Data Analysis

This study employed text mining and SNA to determine the overall characteristics of the Korean exercise healthcare industry. First, TEXTOM and the Netdraw visualization tool of UCINET 6 [28] were used to perform both text mining and SNA.

## 4. Results

### 4.1. Results of the Data Collection

Before the COVID-19 pandemic, the number of data points was 6541, while the data volume was 3053 KB. During the pandemic, the number of data points was 7461, while the volume was 3228 KB. Furthermore, during the pandemic, the datasets were higher and larger than those before the pandemic. In total, 14,002 data points and 6281 KB of data were collected using TEXTOM. Table 2 lists the numbers, data points, and volumes.

### 4.2. Results of Text Mining Analysis

Table 3 presents the results of the frequency analysis of the top 30 terms related to the exercise healthcare industry.

#### 4.2.1. Results of Frequency Analysis

The frequency of terms during the collection period was confirmed via text mining, where the higher a term’s frequency, the more important it is [48]. The frequency analysis with the keywords “exercise”, “healthcare”, and “industry” revealed that before the pandemic, the terms “healthcare” (4041), “industry” (1650), “exercise” (1575), “service” (762), “smart” (611), “education” (603), “characteristics” (555), “health” (537), “technology (520)”, and “fields” (465) appeared in descending order. During the pandemic, the terms “healthcare” (4936), “industry” (2088), “exercise” (1770), “digital” (1286), “service” (1027), “health” (679), “base” (568), “field” (565), “market” (562), and “smart” (546)” appeared in descending order. A comparison of the pre-pandemic and pandemic periods revealed that the terms “service”, “smart”, and “education” were very frequent in the former, whereas the terms “digital”, “service”, and “health” were very frequent in the latter.

#### 4.2.2. Results of TF-IDF Analysis

TF-IDF is a value multiplied by term frequency and IDF, through which the importance of words in the document can be identified, even if certain terms do not appear often [49]. Therefore, the higher the frequency of a word in a specific document and the smaller the number of documents including the word, the higher the TF-IDF value. The TF-IDF analysis revealed that before the pandemic, the terms “industry” (2000.137), “healthcare” (1977.050)” “exercise” (1836.534), “education” (1562.903), “service” (1549.543), “characteristics” (1524.589), “smart” (1440.526), “technology” (1232.047), “health” (1225.286), and “digital” (1196.329) appeared in descending order. During the pandemic, the terms “digital” (2237.458), “industry” (2164.435), “healthcare” (2147.413), “exercise” (1903.085), “service” (1857.953), “health” (1410.802), “smart” (1364.138), “base” (1314.820), “market” (1299.739), and “COVID-19” (1290.155) appeared in descending order. On comparing the pre-pandemic and pandemic periods, we found that the terms “education”, “service”, and “characteristics” were very frequent in the former, whereas the terms “digital”, “service”, and “health” were very frequent in the latter.

### 4.3. SNA Results

#### 4.3.1. Degree Centrality Analysis Results

Table 4 presents the results of the degree centrality analysis of the top 30 terms related to the exercise healthcare industry. Degree centrality analysis can confirm how many relationships a term has with other terms [50]. By linking the exercise healthcare industry with the keywords, the terms “healthcare” (281.102), “industry” (119.593), “exercise” (99.169), “education” (75.136), “characteristics” (72.068), “service” (66.288), “sports” (55.983), “leader” (53.000), “smart” (52.678), and “progress” (52.085) appeared in descending order before the pandemic. During the pandemic, the terms “healthcare” (409.678), “industry” (184.831), “digital” (146.695), “exercise” (140.305), “service” (119.542), “health” (69.627), “offer” (57.458), “market” (55.525), “base” (54.949), and “platform” (54.407) appeared in descending order. A comparison of the pre-pandemic and pandemic periods revealed that the terms “education”, “characteristics”, and “service” were very frequent in the former, whereas the terms “digital”, “service”, and “health” were very frequent in the latter.

#### 4.3.2. CONCOR Analysis Results

Table 5 presents the results of the CONCOR analysis of the top 30 terms related to the exercise healthcare industry. Through the CONCOR analysis, clusters of terms with similarities are derived, through which the entire network structure can be intuitively grasped [51]. Based on the CONCOR analysis of the pre-pandemic period, seven clusters were identified. The first cluster was classified as *smart management*, containing the terms “industry”, “service”, “smart”, “health”, “industrial revolution”, “offer”, “development”, “management”, “business”, and “times”. The second was classified as *future technology*, containing the terms “exercise”, “technology”, “field”, “digital”, “enterprise”, “market”, “treatment”, “domestic”, “uses”, and “future”. The third was classified as *fitness* containing the terms “healthcare” and “fitness”. The fourth was classified as *sports education*, containing the terms “educations” and “sports”. The fifth was classified as *exercise leader* (e.g., personal trainers), containing the terms “characteristics” and “leader”. The sixth was classified as *research*, containing the terms “base” and “research”. The seventh was classified as *rehabilitation*, containing the terms “progress” and “rehabilitations”.

Based on the CONCOR analysis of the pandemic period, seven clusters were identified. The first cluster was classified as *future technology*, containing the terms “industry”, “digital”, “field”, “market”, “enterprise”, “technology”, “treatment”, “future”, and “data”. The second was classified as *smart management*, containing the terms “exercise”, “health”, “smart”, “management”, “uses”, and “individual”. The third was classified as *services*, containing the terms “service”, “platform”, “offer”, “insurance company”, “stay healthy”, and “analysis”. The fourth was classified as *fitness*, containing the terms “healthcare” and “fitness”. The fifth was classified as *business*, containing the terms “base”, “business”, and “development”. The sixth was classified as *COVID-19* and included the terms “COVID-19”, “times”, and “growth”. The seventh was classified as *research*.

Subsequently, *future technology*, *smart management*, *fitness*, and *research* were categorized into similar clusters. In the pre-pandemic period, the clusters of *education*, *exercise leader*, and *rehabilitation* were identified. For the pandemic period, the clusters of *services*, *business*, and *COVID-19* were categorized differently.

**Figure 2:**
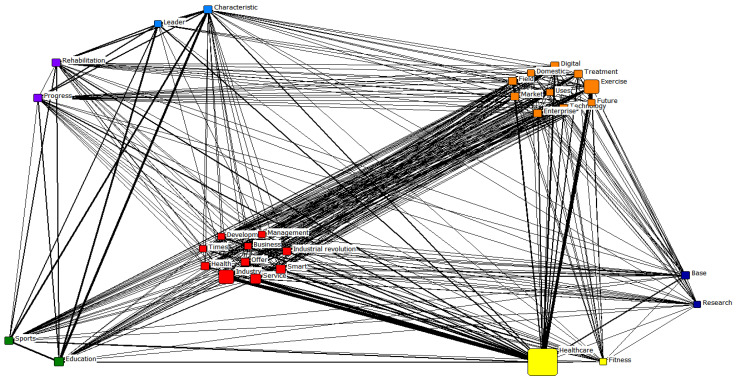
CONCOR analysis results for the pre-pandemic period.

**Figure 3:**
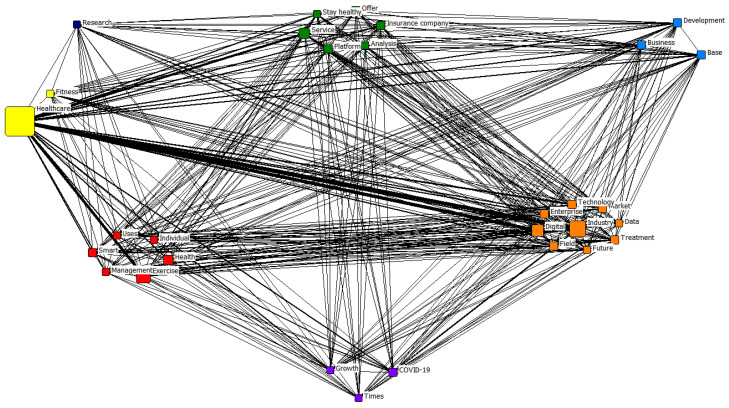
CONCOR analysis results for the pandemic period.

Figure 2 and Figure 3 show the clusters derived from the CONCOR analysis.

## 5. Discussion and Limitations

As the COVID-19 pandemic has changed opportunities and trends in participating in exercise, and digitalization is rapidly occurring due to the spread of non-face-to-face culture, a need exists to analyze the exercise healthcare industry by distinguishing between the pre- and post-COVID-19 periods. According to the Han [24], through big data analysis, it should be approached from the point of view of a complex structure that includes data and the patterns of production and consumption surrounding them, not just as a technical phenomenon in the narrow sense of new data that are different in quantity and quality. Therefore, this study aimed to predict the characteristics of the exercise healthcare industry in the post-pandemic era by comparing the periods before and after the COVID-19 outbreak through big data analysis. As a result of frequency, TF-IDF, and degree centrality analyses, the top 10 derived terms were obtained. Moreover, based on the CONCOR analysis, we determined four similar clusters and six different clusters. Therefore, the first part of the discussion centers on the results of frequency, TF-IDF, and connection centrality analyses, while the second part centers on the CONCOR analysis.

### 5.1. Discussion of Frequency, TF-IDF, and Degree Centrality Analysis

The results of the frequency, TF-IDF, and centrality analyses were similarly derived. To summarize the results, the terms “healthcare”, “industry”, “exercise”, “service”, “smart”, “education”, “characteristics”, “health”, “technology”, and “fields” appeared frequently in the pre-pandemic period. However, during the pandemic period, the terms “healthcare”, “industry”, “exercise”, “digital”, “service”, “health”, “base”, “field”, “market”, and “smart” appeared frequently.

#### 5.1.1. Prior to the Pandemic, Attention as a Tool for Smart Education

In the pre-pandemic period, the exercise healthcare industry attracted attention as a tool for smart education. The feasibility of smart education (e.g., online education and educational methods) had long been the subject of research in the field of education [52], and with lockdowns being issued worldwide due to the COVID-19 pandemic, traditional education modalities were completely converted to online education [52].

The pandemic affected all areas of society [53,54]. Physical activity levels declined significantly during lockdown [55], particularly as facilities such as indoor and outdoor sports facilities and gymnasiums were closed in many countries [56]. Moreover, the non-face-to-face environment naturally expanded into our daily lives [57], where technologies such as ICT platforms served as useful tools during the pandemic period [58].

#### 5.1.2. Focus on Digital Services during the Pandemic

During the pandemic period, people showed increased interest in health through digital services. Thus, “smart” resources used as tools for education have been expanded and applied in the field of health following the outbreak of the pandemic. However, many people experienced difficulties in adapting to online platforms through ICTs (particularly in the early days of the pandemic) but have now become more familiar with them. Furthermore, technologies such as artificial intelligence are attracting the attention of researchers, doctors, technology and program developers, and consumers in various fields due to their potential for transformative innovation in healthcare and public health [59,60,61,62]. Thus, digitalization is progressing rapidly in all fields owing to lockdown measures implemented during the COVID-19 pandemic [63]. However, the exercise healthcare industry has been developing based on ICTs since the Fourth Industrial Revolution, that is, even before the pandemic [28]. In this environment, information is actively generated to provide new digital services, which are becoming a driving force for the development of digital technology. These approaches can contribute toward fulfilling the needs of exercise and healthcare in modern society.

### 5.2. Discussion of CONCOR Analysis

The CONCOR analysis identified the clusters of *smart management*, *future technology*, *fitness*, and *research* as similar clusters. Additionally, the clusters of *education*, *exercise leader*, and *rehabilitation* were derived from the pre-pandemic period, whereas the clusters of *services*, *business*, and *COVID-19* were derived differently for the pandemic period.

#### 5.2.1. The Values That Have Not Changed despite COVID-19

First, *smart management* was identified as a major cluster for both the pre-pandemic and pandemic periods because the exercise healthcare industry aims to provide effective health management services. As public interest in healthcare was high even before the COVID-19 pandemic, awareness in health naturally increased during the pandemic. In addition, more terms clustered during the pandemic compared with the pre-pandemic period because health management campaigns and policies were actively implemented by the government [64]. As the public interest in health increases, the importance of smart health management is emphasized along with the social environment in order to recover from the sedentary habits prevalent during the pandemic.

Second, *future technology* was identified as a major cluster for both the pre-pandemic and pandemic periods. During the COVID-19 pandemic, modern society underwent rapid digitalization based on ICTs [65]. Several advanced technologies have been rapidly implemented in the exercise healthcare industry, especially after the outbreak of COVID-19 [12]. Although the key terms within the cluster are generally similar, considering that the frequency of exposure to digital terms after COVID-19 is high, the range of technology utilization is expected to expand after the pandemic. Despite the difference before and after the pandemic, as the exercise healthcare industry is highly related to the development of science and technology, future technology will provide attributes that will lead to the growth in this sector.

Third, *fitness* was derived as a major cluster for both the pre-pandemic and pandemic periods. Korea’s fitness industry suffered a brief crisis due to the pandemic [66] but is now achieving unprecedented prosperity [67]. Providing customized exercise services for users and more effective health management services are two main aims of the exercise healthcare industry. From this perspective, interest in healthcare through fitness received considerable public attention in both the pre-pandemic and pandemic periods; therefore, this sector can potentially grow into a major industry in the future. Although direct participation in exercise remains the primary method for growth in this industry, various services for fitness healthcare supplemented with ICTs will be developed and provided in the future.

Fourth, *research* was derived as a major cluster for both the pre-pandemic and pandemic periods. The healthcare industry is technology-intensive and should be theoretically supported by advanced ICTs, research on the healthcare industry, effective exercise methods, and investigations on health promotion and management [68,69]. As new studies are published, the healthcare paradigm shifts toward healthcare 3.0 [37]. Therefore, new technologies are expected to develop rapidly in the future, which should be scientifically verified and developed.

#### 5.2.2. Newly Discovered Values Following COVID-19

First, *sports education* was derived as one cluster for the pre-pandemic period; however, it did not form a single cluster for the pandemic period. Korea’s sports education service industry has long provided opportunities for the public to improve their health by participating in sports activities [70]. However, owing to the government’s policy of prohibiting the use of indoor sports facilities during the pandemic period, sports education businesses closed or suffered management difficulties [63]. Thus, public participation in sports was restricted; consequently, the exercise paradigm has changed, with an increase in home training [71]. Therefore, clusters of sports education are no longer formed. In other words, sports education is no longer a field of interest in the exercising healthcare industry.

Second, an *exercise leader* cluster was formed in the pre-pandemic period; however, it did not form a cluster in the pandemic period. Owing to the pandemic, various countries, including Korea, restricted the use of indoor sports facilities to prevent infection, and non-face-to-face exercise environments using online technology expanded [72]. The field of exercise healthcare has also changed from an offline center with exercise leaders (e.g., personal trainers) to online-based platforms such as YouTube [73]. Therefore, the subject that guides exercise is changing from the individual leader to the technological interface.

Third, although it was not so for the pandemic period, *rehabilitation* formed a cluster for the pre-pandemic period. Considering that the exercise healthcare industry aims to provide effective health management services [69], rehabilitation is important. Before the pandemic, the exercise healthcare industry focused on treatment through exercise to promote rehabilitation and health; however, following the outbreak, it changed for the purposes of prevention and health management. Additionally, during the pandemic, clustering for rehabilitation was not achieved because campaigns for the prevention of infectious diseases and health management focused on health prevention. Nevertheless, rehabilitation is a field that must be addressed in the exercise healthcare industry, and the development of rehabilitation-related technology is required in the future.

Fourth, during the pandemic period, *services* appeared as a new cluster. Following the pandemic, digitalization in all fields accelerated because of the spread of an online-oriented, non-face-to-face culture [70]. With the development of online technology, modern society is facing an era of digital transformation, while new exercise healthcare content is continuously being created. Additionally, exercise healthcare programs are expanding based on mobile-device-oriented platforms [11], which will continue to fuel fierce competition among the numerous companies related to exercise healthcare that can immediately provide new services.

Fifth, for the pandemic period, *business* emerged as a new cluster. Even before COVID-19, healthcare services centered on mobile platforms were implemented. However, with the COVID-19 pandemic, the exercise healthcare industry developed rapidly based on mobile platforms [11]. As mobile-related technologies are expected to expand more recently, the diversity of the exercise healthcare industry is also expected to expand. Therefore, the exercise healthcare business field based on mobile platforms is expected to develop further in the future, as the advantages of access are clear.

Finally, *COVID-19* formed a new cluster. Before and after the COVID-19 pandemic, society underwent many changes across various fields [53,54]. Above all, as public interest in health increased due to concerns about infection, public interest in exercise healthcare also increased. The exercise healthcare industry must create more diverse and advanced services to adapt to this social atmosphere. In other words, the pandemic served as an opportunity to suggest a new direction for the growth of the exercise healthcare industry and garner public attention, paradoxically becoming a vehicle for the increasing demand in the exercise healthcare industry.

Nevertheless, this study has several limitations. First, because it analyzed big data with a focus on the Korean context, the findings should be generalized with caution. Second, in the process of collecting and analyzing data, potential biases can arise due to the data not accurately representing the population predicted by the model. Third, as big data analysis examines a vast amount of data, the results may be interpreted differently depending on the researcher’s viewpoint. Particularly, unlike previous studies, big data research may be limited to forecasting purposes.

## 6. Conclusions

This study compared and analyzed data before and after the COVID-19 pandemic using the keywords “exercise”, “healthcare”, and “industry”. We identified the top 30 terms using a vast amount of data from social media. Four common clusters (*smart management*, *future technology*, *fitness*, and *research*) and six different clusters (*sports education*, *exercise leader*, *rehabilitation*, *services*, *business*, and *COVID-19*) were derived by comparing data for both the pre-pandemic and pandemic periods. *Smart management*, *future technology*, *fitness*, and *research* remained important in the exercise and healthcare industry across both periods. Additionally, during the COVID-19 pandemic, *services*, *business*, and *COVID-19* emerged as new values. The results of this study are significant and can influence future research and the development of exercise healthcare techniques. The results provide meaningful data and offer valuable insights to explore the changing trends in exercise healthcare. We expect significant implications for future value creation in related fields to be derived through data analysis over time.

## Figures and Tables

**Table 1:** Data collection procedure.

Category	Content
Collection channel	Naver, Google
Collection period	Pre-pandemic period: 1 January 2018–31 December 2019 Pandemic period: 1 January 2020–31 December 2021
Collection tool	TEXTOM (The IMC Inc., Daegu, Republic of Korea) (http://textom.co.kr, (accessed on 1 March 2022))
Search keywords	Exercise, Healthcare, Industry

**Table 2:** Collection channels, number of data points, and volume.

Period	Number of Data Points	Volume
Pre-pandemic	6541	3053 KB
Pandemic	7461	3228 KB
Total	14,002	6281 KB

**Table 3:** Results of text mining analysis.

	Pre-Pandemic Period		Pandemic Period		Pre-Pandemic Period		Pandemic Period	
	Frequency Analysis				TF-IDF Analysis			
Rank	Term	Freq.	Term	Freq.	Term	Freq.	Term	Freq.
1	Healthcare	4041	Healthcare	4936	Industry	2000.137	Digital	2237.458
2	Industry	1650	Industry	2088	Healthcare	1977.050	Industry	2164.435
3	Exercise	1575	Exercise	1770	Exercise	1836.534	Healthcare	2147.413
4	Service	762	Digital	1286	Education	1562.903	Exercise	1903.085
5	Smart	611	Service	1027	Service	1549.543	Service	1857.953
6	Education	603	Health	679	Characteristics	1524.589	Health	1410.802
7	Characteristics	555	Base	568	Smart	1440.526	Smart	1364.138
8	Health	537	Field	565	Technology	1232.047	Base	1314.820
9	Technology	520	Market	562	Health	1225.286	Market	1299.739
10	Field	465	Smart	546	Digital	1196.329	COVID-19	1290.155
11	Digital	460	COVID-19	544	Leader	1171.704	Field	1271.861
12	Progress	455	Enterprise	530	Sports	1148.222	Technology	1251.037
13	Sports	441	Technology	523	Field	1119.536	Enterprise	1232.513
14	Enterprise	440	Platform	502	Enterprise	1102.142	Platform	1218.379
15	Industrial revolution	433	Offer	499	Market	1089.127	Insurance company	1185.656
16	Market	432	Treatment	451	Industrial revolution	1070.860	Offer	1182.312
17	Base	402	Business	451	Progress	1051.086	Treatment	1163.943
18	Offer	390	Development	435	Base	1044.300	Business	1118.805
19	Rehabilitation	386	Insurance company	424	Treatment	1027.740	Development	1108.425
20	Treatment	382	Management	397	Offer	1027.182	Research	1077.588
21	Leader	367	Uses	387	Research	981.396	Management	1040.282
22	Development	353	Research	366	Rehabilitation	973.155	Uses	997.701
23	Fitness	348	Stay healthy	349	Fitness	957.152	Stay healthy	956.114
24	Research	317	Times	341	Development	944.052	Times	945.453
25	Management	308	Growth	333	Business	892.237	Fitness	936.475
26	Business	307	Fitness	332	Management	871.347	Growth	918.832
27	Domestic	294	Future	319	Mobile	855.310	Data	898.506
28	Times	293	Individual	308	Uses	846.907	Future	895.344
29	Uses	291	Data	306	Times	841.145	Individual	893.612
30	Future	286	Analysis	299	Domestic	835.038	Artificial intelligence	887.761

Note: TF-IDF, term frequency–inverse document frequency analysis.

**Table 4:** Results of degree centrality analysis.

	Pre-Pandemic Period		Pandemic Period	
Rank	Term	Freq.	Term	Freq.
1	Healthcare	281.102	Healthcare	409.678
2	Industry	119.593	Industry	184.831
3	Exercise	99.169	Digital	146.695
4	Education	75.136	Exercise	140.305
5	Characteristics	72.068	Service	119.542
6	Service	66.288	Health	69.627
7	Sports	55.983	Offer	57.458
8	Leader	53.000	Market	55.525
9	Smart	52.678	Base	54.949
10	Progress	52.085	Platform	54.407
11	Rehabilitation	49.458	Smart	54.051
12	Technology	44.492	Technology	53.644
13	Digital	43.915	Field	52.559
14	Health	40.712	Insurance company	49.407
15	Field	37.610	Treatment	49.305
16	Industrial revolution	36.881	Enterprise	44.763
17	Base	34.000	Uses	43.254
18	Offer	33.322	Management	42.508
19	Market	32.898	Development	41.983
20	Enterprise	32.559	Business	40.254
21	Treatment	31.780	Stay healthy	38.763
22	Development	27.661	COVID-19	38.492
23	Uses	24.254	Growth	37.169
24	Management	23.797	Data	37.102
25	Times	23.627	Individual	35.763
26	Fitness	23.271	Analysis	33.712
27	Future	22.559	Times	31.729
28	Business	20.763	Fitness	30.542
29	Domestic	20.661	Future	30.237
30	Research	16.136	Research	19.746

**Table 5:** Results of CONCOR analysis.

	Cluster	Terms	Freq.
	Pre-pandemic period		
1	Smart management	Industry, service, smart, health, industrial revolution, offer, development, management, business, and times	10
2	Future technology	Exercise, technology, field, digital, enterprise, market, treatment, domestic, uses, and future	10
3	Fitness	Healthcare and fitness	2
4	Sports education	Education and sports	2
5	Exercise leader	Characteristics and leader	2
6	Research	Base and research	2
7	Rehabilitation	Progress and rehabilitation	2
	Pandemic period		
1	Future technology	Industry, digital, field, market, enterprise, technology, treatment, future, and data	9
2	Smart management	Exercise, health, smart, management, uses, and individual	6
3	Services	Service, platform, offer, insurance company, stay healthy, and analysis	6
4	Fitness	Healthcare and fitness	2
5	Business	Base, business, and development	3
6	COVID-19	COVID-19, times, and growth	3
7	Research	Research	1

## Data Availability

Not applicable.
